# One Week of CDAHFD Induces Steatohepatitis and Mitochondrial Dysfunction with Oxidative Stress in Liver

**DOI:** 10.3390/ijms22115851

**Published:** 2021-05-29

**Authors:** Takehito Sugasawa, Seiko Ono, Masato Yonamine, Shin-ichiro Fujita, Yuki Matsumoto, Kai Aoki, Takuro Nakano, Shinsuke Tamai, Yasuko Yoshida, Yasushi Kawakami, Kazuhiro Takekoshi

**Affiliations:** 1 Laboratory of Laboratory/Sports Medicine, Division of Clinical Medicine, Faculty of Medicine, University of Tsukuba, 1-1-1 Tennodai, Tsukuba 305-8577, Ibaraki, Japan; take0716@krf.biglobe.ne.jp (T.S.); yonamine.masato.fu@u.tsukuba.ac.jp (M.Y.); shin.ichiro.fujita.03@gmail.com (S.-i.F.); K-Aokitsuku@md.tsukuba.ac.jp (K.A.); y-yoshida@tius.ac.jp (Y.Y.); y-kawa@md.tsukuba.ac.jp (Y.K.); 2 Master’s Program in Medical Sciences, Graduate School of Comprehensive Human Sciences, University of Tsukuba, 1-1-1 Tennodai, Tsukuba 305-8577, Ibaraki, Japan; s2021387@s.tsukuba.ac.jp (S.O.); s2021408@s.tsukuba.ac.jp (T.N.); 3 Research and Development Section, Anicom Specialty Medical Institute Inc., 2-6-3 Chojamachi 5F, Yokohamashi-Nakaku 231-0033, Kanagawa, Japan; ymatsumoto.ac@gmail.com; 4 Japan Society for the Promotion of Science, Kojimachi Business Center Building, Kojimachi, Chiyoda-ku, Tokyo 102-0083, Japan; 5 Doctoral Program in Sports Medicine, Graduate School of Comprehensive Human Sciences, University of Tsukuba, 1-1-1 Tennodai, Tsukuba 305-8577, Ibaraki, Japan; tama1994@outlook.jp; 6 Department of Medical Technology, Faculty of Health Sciences, Tsukuba International University, 6-20-1 Manabe, Tsuchiura 300-0051, Ibaraki, Japan

**Keywords:** CDAHFD, NASH, mitochondrial dysfunction, liver, oxidative stress

## Abstract

The prevalence of nonalcoholic fatty liver disease (NAFLD) has been rapidly increasing worldwide. A choline-deficient, L-amino acid-defined, high-fat diet (CDAHFD) has been used to create a mouse model of nonalcoholic steatohepatitis (NASH). There are some reports on the effects on mice of being fed a CDAHFD for long periods of 1 to 3 months. However, the effect of this diet over a short period is unknown. Therefore, we examined the effect of 1-week CDAHFD feeding on the mouse liver. Feeding a CDAHFD diet for only 1-week induced lipid droplet deposition in the liver with increasing activity of liver-derived enzymes in the plasma. On the other hand, it did not induce fibrosis or cirrhosis. Additionally, it was demonstrated that CDAHFD significantly impaired mitochondrial respiration with severe oxidative stress to the liver, which is associated with a decreasing mitochondrial DNA copy number and complex proteins. In the gene expression analysis of the liver, inflammatory and oxidative stress markers were significantly increased by CDAHFD. These results demonstrated that 1 week of feeding CDAHFD to mice induces steatohepatitis with mitochondrial dysfunction and severe oxidative stress, without fibrosis, which can partially mimic the early stage of NASH in humans.

## 1. Introduction

Nonalcoholic fatty liver disease (NAFLD) includes simple steatosis (nonalcoholic fatty liver: NAFL) and nonalcoholic steatohepatitis (NASH). NASH can be progressive, and predisposes individuals to the development of fibrosis and cancer [1]. Moreover, unlike other organs, it is well-known that many types of liver damage, such as simple steatosis, steatohepatitis, and fibrosis, are unlikely to cause specific subjective symptoms, although the patients may have systematic symptoms that are non-specific to the damage [2,3]. Additionally, NAFLD is rapidly becoming the most common cause of chronic liver disease, due to an increase in the prevalence of obesity in recent years [4]. For the same reason, the incidence and prevalence of NAFLD are rapidly increasing worldwide [1]. Therefore, the development of new treatments, particularly therapeutic medicines that can be used to treat patients with early-stage NAFLD, has become urgent. However, no medicines have yet been approved to treat NAFLD [5].

There are many studies using animal models of NAFLD to develop new treatments and to investigate its molecular mechanisms. In general, the NAFLD model is created by an unbalanced diet, such as a high-fat diet (HFD) [6,7,8], a high-sucrose high-fat diet (HS-HFD) [9,10], or a high-fat high-cholesterol diet [11,12]. Moreover, NASH models including fibrosis and steatohepatitis have been made using a choline-deficient, L-amino acid-defined, high-fat diet (CDAHFD) [13,14,15,16]. The animal experiments using the various models mentioned above lasted 8 to 12 weeks or longer [6,7,8,9,10,11,12,13,14,15,16]. Such long-term experiments can sometimes be a major impediment to the progress of research, as it takes a very long time to obtain results. In addition, most public grants are time-limited, and the researcher must complete the experiment as soon as possible and move on to the next steps. Considering these factors, time-saving animal models are extremely valuable because they accelerate research.

Given that CDAHFD progresses faster to liver steatosis than HFD, it can be used to create a more severe steatohepatitis with fibrosis (NASH with fibrosis) model of the liver. This is because the formulation of the diet as choline-deficient, L-amino acid-defined restricts the secretion of triglycerides (TG) into the blood from the liver. Therefore, we hypothesized that even a short feeding period, such as 1 week, may induce simple fatty liver or steatohepatitis that resembles NAFL or NASH. Additionally, in our experimental experience, we preliminarily observed a fatty liver after only 1 week of feeding CDAHFD. In addition, to date, there has been no report with a detailed analysis at the molecular level of the liver after 1 week of feeding this diet, and the influences of the diet are unknown.

Therefore, this study aimed to investigate and clarify in detail the influence of 1 week of CDAHFD feeding on the liver. Moreover, we focused not only on parameters that reflect a fatty liver but also on parameters of inflammation, mitochondrial dysfunction, and oxidative stress, because these three parameters are strongly associated with the progression of the disease state in NAFLD [17,18,19], according to the second hit theory [20,21]. 

In the results, we observed a similar pathology to early-stage NASH without fibrosis in the model. Furthermore, we provide several important evaluation methods of the model. We expect these contributions will accelerate the research progress.

## 2. Results

### 2.1. Phenotypic Analyses Showed Significant Changes in Liver in CDAHFD Group

**Figure 1:**
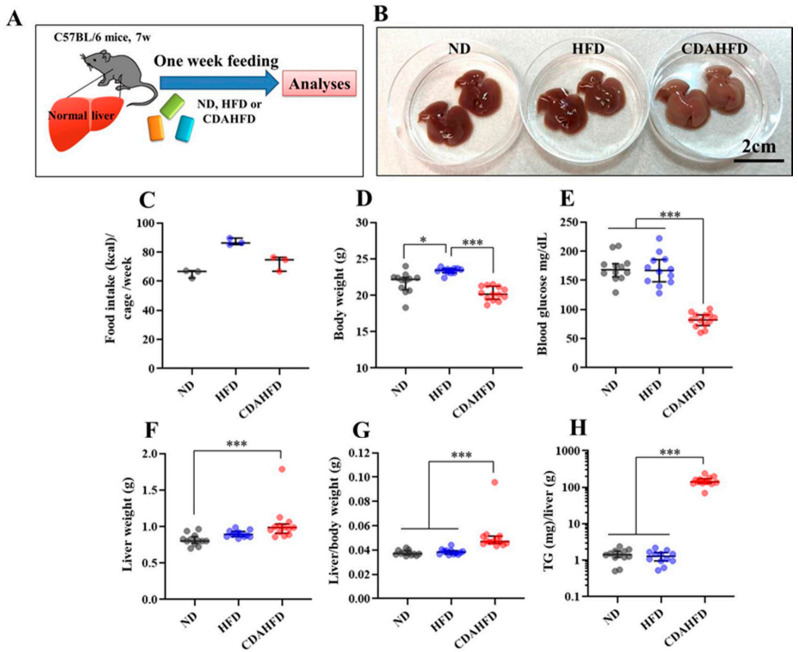
Results of phenotypic analyses. (**A**) Overview of animal experiment, (**B**) exterior photographs of liver of representative samples in each group, (**C**) food intake (kcal)/cage/week, each group had three cages (three plots) including three to five mice each, (**D**) body weight (g), (**E**) blood glucose (mg/dL), (**F**) liver weight (g), (**G**) liver/body weight (g), (**H**) TG (mg)/liver (g). * *p* < 0.05, *** *p* < 0.001, respectively.

The overview of this animal experiment is shown in Figure 1A. Seven-week-old mice were randomly assigned to three groups and fed a normal (ND; *n* = 12), an HFD (*n* = 12), or a CDAHFD (*n* = 13) diet for 1 week. In the CDAHFD group, exterior photographs of the liver at dissection showed significant changes from brown-red to brown-white (Figure 1B), which may reflect lipid accumulation. Food intake (kcal)/cage/week was slightly higher in the HFD and CDAHFD groups than in the ND group (Figure 1C). Body weight slightly increased in the HFD group only (Figure 1D). Blood glucose in the fasting condition for 5 h was significantly decreased in the CADHFD group compared to the other two groups (Figure 1E). The liver weight and liver/body weight were slightly increased in the CDAHFD group compared with the other two groups (Figure 1F,G). The amount of TG in the liver tissue was drastically increased in the CDAHFD group, approximately 100-fold higher than the other two groups. These results suggest that mice fed a CDAHFD for 1 week can develop a severely fatty liver despite lowered blood glucose levels.

### 2.2. CDAHFD Generated Lipid Droplets in Liver with Inflammatory Response 

**Figure 2:**
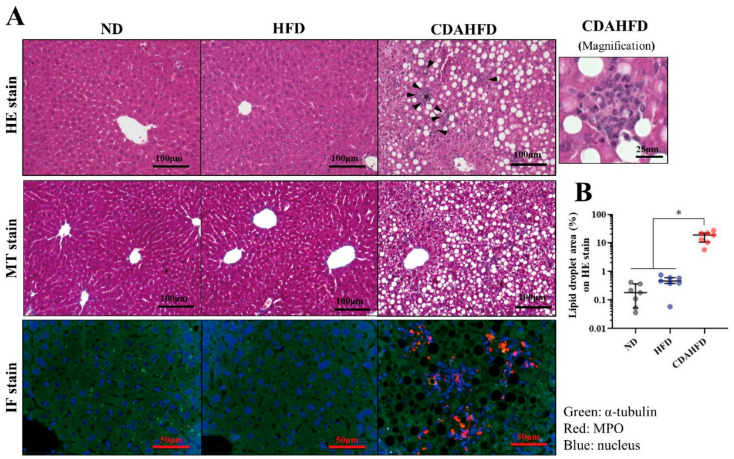
Morphological observation of liver after ND, HFD, or CDAHFD. (**A**) HE, MT, and IF staining for liver in each group. The site of cell accumulation observed on the HE stain is individually magnified in the right figure. (**B**) Quantifications of the lipid droplet areas (%) on the HE staining for 7 samples in each group. * *p* < 0.05.

Tissue slides of formalin-fixed paraffin-embedded (FFPE) samples subjected to staining and morphologic analyses were created using seven randomly selected samples from each group. The results of the tissue staining are shown in Figure 2A. In the hematoxylin–eosin (HE) and Masson’s trichrome (MT) staining, many lipid droplets were observed in the CDAHFD group (Figure 2A (HE) and (MT) staining). Supporting these observations, the lipid droplet areas (%) on the HE-stained slides were drastically increased in the CDAHFD group compared to the other two groups (Figure 2B). On the other hand, no fibrosis was observed by MT staining (Figure 2A (MT) staining).

As shown by the arrowheads, cell accumulations were often observed in the CDAHFD group on the HE stains. The asterisk site is magnified on the right side, and the accumulated cells appeared to be inflammatory cells, such as neutrophils that have a characteristic morphology with a lobed nucleus (Figure 2A (HE) stains). In support of these observations, immunofluorescence (IF) staining revealed the localization of myeloperoxidase at the cell accumulation site (Figure 2A (IF) stain). Thus, it was concluded that neutrophils were the main component of the cell accumulations.

In addition, the degrees of pathological progression were evaluated using the Steatosis, Activity, and Fibrosis (SAF) scoring system [22]. The results indicated that all samples in the CDAHFD group were score 4 (steatosis: 1, portal inflammation: 2, ballooning: 1, and fibrosis: 0, in all samples), and all samples in the other two groups were score 0.

These results suggest that one week of feeding a CDAHFD induces the deposition of lipid droplets with infiltration of inflammatory cells without fibrosis, which seems to be early-stage NASH.

### 2.3. CDAHFD Increased Liver Deviation-Enzyme Activities in Plasma

**Figure 3:**
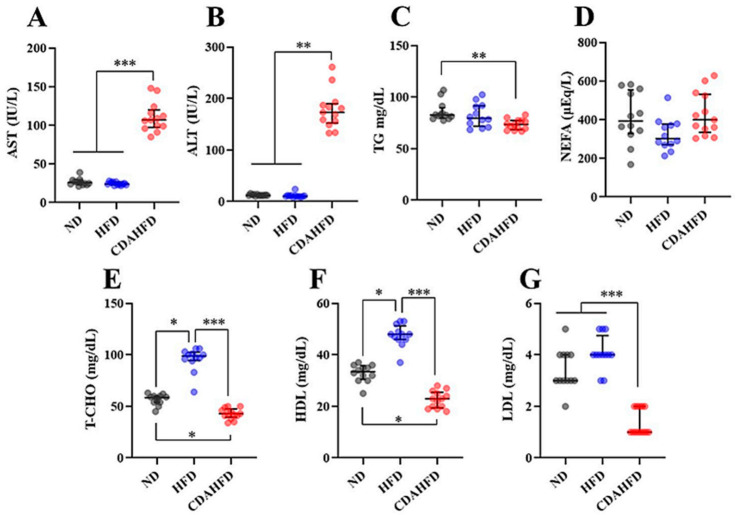
Measurements of general biomarkers in plasma. (**A**) AST (IU/L), (**B**) ALT (IU/L), (**C**) TG (mg/dL), (**D**) NEFA (µEq/L), (**E**) T-CHO (mg/dL), (**F**) HDL (mg/dL), (**G**) LDL (mg/dL). * *p* < 0.05, ** *p* < 0.01, *** *p* < 0.001, respectively.

General biomarkers were measured in the plasma from the mice. The activities of aspartate transaminase (AST) and alanine aminotransferase (ALT) were significantly increased in the CDAFD group compared to in the other two groups (Figure 3A,B). In particular, the ALT activity of the CDAHFD group was 14- to 16-fold higher than that of the other two groups (Figure 3B). Plasma TG was slightly decreased in the CDAHFD group compared to the control group (Figure 3C). The non-esterified free fatty acid (NEFFA) was not significantly altered (Figure 3D). Total cholesterol (T-CHO) and low/high-density lipoprotein cholesterol (HDL/LDL) were significantly decreased in the CDAHFD group compared to the other groups (Figure 3E–G). On the other hand, T-CHO and HDL were significantly increased in the HFD group compared to the other two groups (Figure 3E,F). These results suggest that 1 week of feeding a CDAHFD rapidly induced liver damage, despite lowered plasma cholesterol.

### 2.4. CDAHFD Increased Oxidative Stress and Mitochondrial Dysfunction in Liver

**Figure 4:**
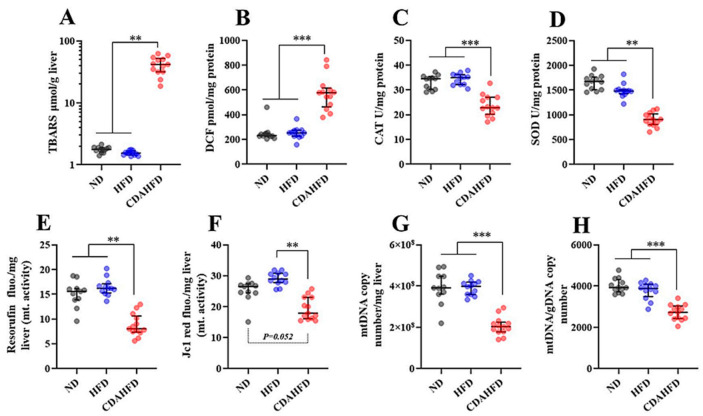
Measurements of oxidative stress markers, antioxidant activities, mitochondrial activities, and copy numbers in liver tissue. (**A**) TBARS (µmol/g liver), (**B**) DCF (pmol/mg protein), (**C**) CAT activities (U/mg protein), (**D**) SOD activities (U/mg protein). (**E**) Resorufin fluorescence/mg liver of Resazurin assay, which can measure mitochondrial activity, (**F**) JC-1 red fluorescence mg liver of JC-1 assay, which can also measure mitochondrial activity, (**G**) mtDNA copy numbers/mg liver in mitochondrial fraction, (**H**) mtDNA/gDNA copy numbers in whole liver tissue. ** *p* < 0.01, *** *p* < 0.001, respectively.

Oxidative stress markers, antioxidant enzyme activity, mitochondrial activity, and DNA copy numbers were measured in the liver tissue. In the results, thiobarbituric acid reactive substance (TBARS) and dichlorofluorescein (DCF), a parameter of lipid peroxidation and reactive oxygen species (ROS), were significantly increased in the CDAHFD group compared to in the other two groups (Figure 4A,B). In particular, TBARS in the CDAHFD group was 23- to 26-fold higher than that in the other two groups (Figure 4A). On the other hand, catalase (CAT) and superoxide dismutase, as first-line antioxidants, were significantly decreased in the CDAHFD group compared to in the other two groups (Figure 4C,D). Moreover, Resazurin and JC-1 assays, which can reflect mitochondrial activity, indicated significantly or marginally decreased activity in the CDAHFD group compared to in the other two groups (Figure 4E,F). Consistent with this finding, mitochondrial DNA (mtDNA) copy numbers in the mitochondrial fraction and the whole liver were significantly decreased (Figure 4G,H). These results suggest that 1 week of feeding a CDAHFD increased oxidative stress and mitochondrial dysfunction with decreased antioxidants in the liver and inflicted damage on the mitochondria by severe lipid peroxidation reaction.

### 2.5. CDAHFD Decreased Mitochondrial Proteins and Increased NRF2 Target Proteins in Liver

**Figure 5:**
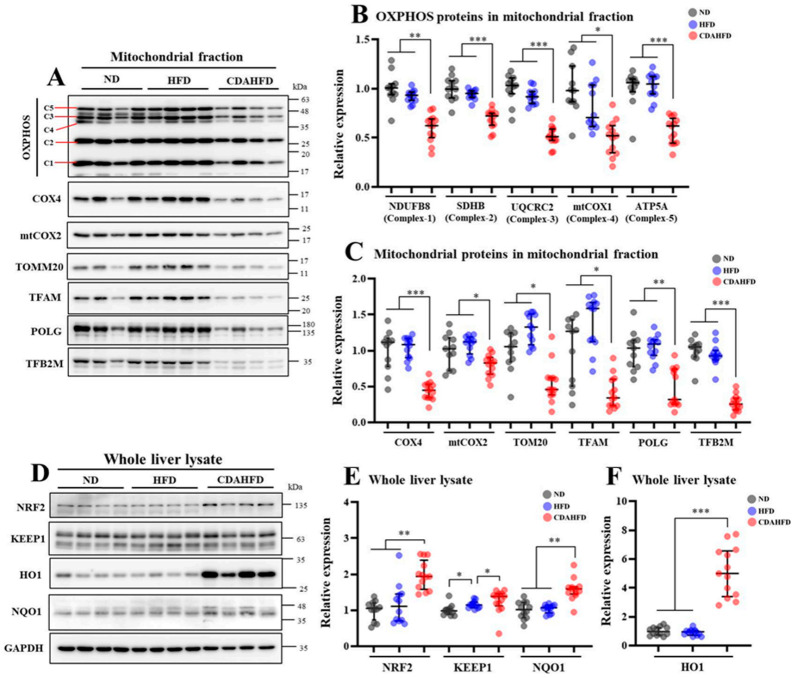
Measurements of mitochondrial proteins in mitochondrial fraction and NRF2 target proteins in whole liver lysate on WB analysis. (**A**) Representative blot data of mitochondrial proteins in isolated mitochondrial fraction as *n* = 4 in each group, C1–5 denote mitochondrial complexes 1–5, (**B**) relative protein expression in quantified results for band of OXPHOS protein in all samples, (**C**) relative protein expression in quantified results for bands of other mitochondrial proteins for all samples, (**D**) representative blot data of NRF2 target proteins in whole liver lysate as *n* = 4 in each group, (**E**,**F**) relative protein expression in quantified results for a band of NRF2 for all samples. * *p* < 0.05, ** *p* < 0.01, *** *p* < 0.001, respectively.

Oxidative phosphorylation (OXPHOS) proteins (complex proteins) and other constituent proteins in the mitochondria were quantified using an isolated mitochondrial fraction in Western blot (WB) analysis. Nuclear factor erythroid 2-related factor 2 (NRF2) target proteins were also quantified using whole liver protein, which reflects oxidative stress. In the blot data in Figure 5A that show representative samples with *n* = 4 in each group, it seemed that OXPHOS and other constituent proteins in the mitochondria were decreased. Supporting this result, the quantitative values of the protein expression levels of OXPHOS and other constituent proteins of the mitochondria were significantly decreased in the CDAHFD group compared to the other two groups (Figure 5B,C). In the blot data in Figure 5D, it seemed that NRF2 target proteins were increased. Supporting this result, the quantitative values of NRF2, NQO1, and HO1 were significantly increased compared to the other two groups (Figure 5E,F). These results suggest that 1 week of feeding a CDAHFD induces decreases in the expression of mitochondrial proteins with activation of NFRs by oxidative stress.

### 2.6. CDAHFD Modified Gene Expression Patterns for Inflammation, Fibrosis, Oxidative Stress, Lipogenesis, and Gluconeogenesis in Liver, with Upregulated Inflammatory Marker Genes in Whole Blood

**Figure 6:**
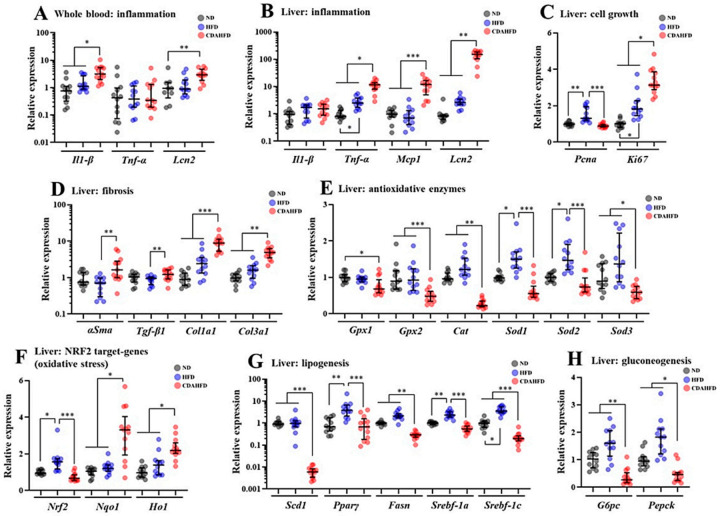
Quantification of gene expression for several marker genes in whole blood and liver. The types of samples are whole blood (**A**) and liver (**B**–**H**). (**A**) Inflammatory markers in whole blood, (**B**) inflammatory markers in liver, (**C**) cell growth markers in liver, (**D**) fibrosis markers in liver, (**E**) antioxidative enzymes in liver, (**F**) NRF2 target genes in liver, (**G**) lipogenesis markers in liver, (**H**) gluconeogenesis markers in liver. * *p* < 0.05, ** *p* < 0.01, *** *p* < 0.001, respectively.

The expression of genes involved in the inflammatory response was measured in the whole blood. *Il1-β* and *Lcn2*, reflecting the acute inflammatory response, were significantly increased in the CDAHFD group compared to the other groups, although the expression of *Tnf-α* was not significantly changed (Figure 6A). The expression of each marker gene for inflammation, cell growth, fibrosis, antioxidant enzymes, NFR2 target genes (response to oxidative stress), lipogenesis, and gluconeogenesis was measured using liver tissues. The inflammation marker genes such as *Tnf-α*, *Mcp1,* and *Lcn2* were drastically increased in the CDAHFD group compared to the other two groups (Figure 6B). In particular, *Lcn2* was the most increased by 182-fold in the CDAHFD group compared to in the ND group (Figure 6A). In the cell growth markers, *Ki67* was significantly increased in the CDAFHD group compared to in the other two groups (Figure 6C). In the cell fibrosis markers, gene expression of *Col1a1* and *Col3a1* was significantly increased in the CDAHFD group compared to the other two groups (Figure 6D). In the markers of antioxidant enzymes, the gene expression of *Gpx2*, *Cat,* and *Sod3* was significantly decreased in the CDAHFD group compared to in the other two groups. Gene expression of *Gpx1* was decreased in the CDAHFD group only when compared to the expression in the ND group. Gene expression of *Sod1* and *Sod2* was decreased in the CDAHFD group compared to in the HFD group (Figure 6E). When the integrated analyses for *Sod1* and *Sod2* in each group were performed, the values were significantly decreased in the CDAHFD group compared to the other two groups, even though they were significantly increased in the HFD group compared to in the other two groups (Supplementary Figure S1). In the NRF2 target genes, the gene expression of *Nqo1* and *Ho1* was significantly increased in the CDAHFD group compared to the expressions in the other two groups, although Nrf2 was increased in the HFD group compared to in the other two groups (Figure 6F). In the marker genes of lipogenesis, the gene expression of *Scd1*, *Fasn*, and *Srebf-1c* was significantly decreased in the CDAHFD group compared to in the other two groups (Figure 6G). In particular, *Scd1* was drastically decreased by 151-fold in the CDAHFD group compared to in the ND group (Figure 6G). On the other hand, the gene expressions of *Pparγ* and *Srebf-1a* in the ND and CDAHFD groups showed no difference (Figure 6G). In the marker genes of gluconeogenesis, the gene expression of *G6pc* and *Pepck* was significantly decreased in the CDAHFD group compared to in the other two groups (Figure 6G). From these results, the CDAHFD appears to induce severe inflammation, increased fibrosis, decreased antioxidant enzymes, increased oxidative stress, decreased de novo lipogenesis, and decreased gluconeogenesis in the liver tissue.

## 3. Discussion

The number of patients affected by NAFLD (including NASH) has been rapidly increasing worldwide, and the disease predisposes individuals to develop fibrosis and liver cancer [1]. Therefore, it is important to understand the molecular mechanism of NAFLD, including NASH, to develop new treatments. Understanding the stage of NASH before the development of fibrosis can be particularly useful in stopping its progression to irreversible NAFLD. Therefore, we tried to establish a mice model of early-stage NASH by feeding the mice a CDAHFD for 1 week. The mice developed liver damage with hepatic steatosis and elevated hepatic TG, which is associated with elevated liver damage markers in the plasma. On the other hand, the mice that were fed a HFD were not observed to have liver damage or lipid deposition. Furthermore, we confirmed that the inflammatory response and oxidative stress in the liver were significantly increased in only the CDAHFD group. Lipid peroxidation in particular was drastically increased upon feeding the mice a CDAHFD for 1 week. Additionally, our results showed that the mitochondrial function and mitochondrial numbers and proteins in the liver were significantly decreased in the CDAHFD group, which suggests that the mitochondria suffer severe damage—this phenomenon is also similar to that observed in NASH patients [23]. 

In this model, we observed severe damage with mitochondrial dysfunction in mice livers. Conversely, the pathological findings showed no fibrosis, even though the gene expression of fibrosis markers was significantly increased. Given the above in an integrated manner, it is considered that the NASH model can be established in only 1 week, and this model is before fibrosis develops, with inflammation, oxidative stress, and mitochondrial damage. 

We also performed a human-adapted pathological examination for the liver in this model. The Steatosis, Activity, and Fibrosis (SAF) scoring system has been used globally as an indicator to assess the progression in the liver of NAFLD on clinical examinations of humans. The SAF score can indicate steatosis, lobular inflammation, portal inflammation, ballooning, and fibrosis using pathological specimens [22]. This scoring system can also be applied to animal models of NAFLD [24]. Therefore, we applied the SAF scoring system for the pathological specimens of the model. The results showed that all samples in the CDAHFD group were score 4 (steatosis: 1, portal inflammation: 2, ballooning: 1, and fibrosis: 0, in all samples). In contrast, all samples in the other two groups had a score of 0. The SAF score of the CDAHFD-fed group proves that the liver phenotype of this model is not simple steatosis (NAFL) but active and progressive NASH [22]. Histological activity in SAF scores is also known to be related to the severity of fibrosis [25]. Therefore, it can be judged that the pathological condition of this model is NASH without fibrosis, with active and progressive advancement of the pathology. In this way, even when the pathological specimen of this model is evaluated in the same way humans are, it can be reliably considered to be early NASH without fibrosis, which supports the results of the other assays. 

This model, which is created in just 1 week, would be highly useful in studies of NAFLD, including NASH. Researchers using this model can save a significant amount of time on NAFLD research. Many studies using NAFLD models have reported requiring long-term feeding periods of 8 to 12 or more weeks [6,7,8,9,10,11,12] to establish the model, which results in a significant loss of time. This model, however, progresses in pathology faster than a normal HFD, even though it is able to be established faster, so if researchers want to develop a drug with this model, they may require a stronger efficacy. Therefore, researchers may need to fully understand the characteristics of each model before conducting experiments. In summary, the main feature of this model is an early-stage model of NASH without fibrosis and it is useful for establishing a therapeutic strategy before the condition causes fibrosis.

The progression of pathogenesis in NAFLD, including NASH, at the cellular level with a focus on mitochondria has been understood as follows. Fatty acid accumulation occurs due to an increase in the influx of fatty acids into the liver by excessive dietary fat [26]. Then, the excessive flux of fatty acids into the mitochondria of the hepatocytes induces excessive oxidative stress, followed by oxidative stress that leads to apoptosis and necrosis of the cells [26]. When mitochondria experience damage, mitochondrial activity and ATP synthesis are attenuated [27]. In addition, excessive oxidative stress causes necrotic inflammation of hepatocytes [28]. Phenomena that are consistent with the above-mentioned mechanisms were observed in our model as follows. The intensities of the Resorufin and JC-1-red fluorescence, which can reflect mitochondrial activity and number, were significantly decreased in the livers of the CDAHFD group compared to the other two groups. Additionally, the mitochondrial complex and components and the mitochondrial DNA copy number were also significantly decreased in the livers of the CDAHFD group. Moreover, the gene expression of inflammatory markers, particularly neutrophil markers that indicate acute inflammation, were drastically increased in the livers of the CDAHFD group, and we also observed neutrophil infiltration and accumulation on the pathological specimens. Therefore, we can explain that our model of 1-week CDAHFD feeding induces mitochondrial damage with excessive fatty acid flux to the mitochondria, accompanied by severe oxidative stress and necrotic inflammation to the hepatocytes, thus forming a vicious cycle. Taken together with the mechanisms of NAFLD and the results of our experiments, it can be declared that our model has the same pathogenesis of NAFLD. Speculating from these results, the hepatocytes in the CDAHFD group may suffer energy metabolism disability with disrupted ATP production and may enter a further vicious cycle due to ATP depletion. However, this study did not focus on ATP production. The amount of ATP produced in liver tissue would also be an important parameter to evaluate when conducting experiments using this model.

Antioxidant dynamics is also considered an important parameter in NAFLD because previous studies of NAFLD patients revealed that the activities of some antioxidant enzymes were significantly decreased in the livers of these patients, with increased oxidative stress [29]. Our results indicated that parameters that reflect oxidative stresses such as TBARS and DCF were significantly increased in the livers of the CDAHFD group compared to the other two groups. On the other hand, gene expressions of *Gpx*s (glutathione peroxidases), *Cat* (catalase), and *Sod*s (superoxide dismutases) were significantly decreased in the CDAHFD group. In contrast, the protein and gene expression of *Ho1* (hem oxinase1), an antioxidant enzyme that is an NFR2 target, were significantly increased in the CDAHFD group compared to in the other two groups. According to measurements of enzyme activity in the liver, CAT and SOD activities were significantly decreased in the CDAHFD group. GPX, SOD, and CAT play a very important role as antioxidants in the first line of defense against ROS to prevent oxidative stress [30]. Therefore, it can be inferred that the antioxidant capacity in the liver was significantly impaired for the CDAHFD group, associated with drastically increased lipid peroxidation, and this canceled the antioxidant effect of the HO1. In addition, these results of the model are consistent with the analysis results of clinical samples of the human liver [29]. Therefore, because the model has similar phenomena to the dynamics of antioxidants in human NAFLD, it can be recognized as an important reason for explaining the usefulness of this model. Although the enzyme activity of GPX was not measured in this study, it can be strongly inferred that it will be a useful evaluation parameter when using this model. In future studies, it is strongly recommended that the enzymatic activity of GPX also be measured.

It has been reported that NASH patients suffer mitochondrial dysfunction associated with decreasing enzyme activities of the mitochondria respiratory chain (MRC) complexes (also called the OXPHOS protein) in the liver [23]. Moreover, several review articles have supported the existence of mitochondrial dysfunction in NASH patients [31,32,33]. An abnormality of the mitochondria causes the function and components of the respiratory chain complex and electron transfer efficiency to degrade, resulting in increased ROS generation and causing oxidative stress [23,34]. In addition, oxidative stress causes mitochondrial damage [33,35]. Therefore, maintaining or amplifying mitochondrial function along with defending against oxidative stress may be beneficial as a treatment for NASH patients. In this study, we established a NASH mouse model with accompanying mitochondrial dysfunction and severe oxidative stress that mimic the NASH condition in humans. We also established an evaluation method of the characteristics of the model. Therefore, this model and the evaluation methods in this study can be expected to be useful for the development of new treatments for humans, focusing on antioxidants and the maintenance of mitochondrial function. For example, applying compounds with antioxidant and mitochondrial activation abilities, such as resveratrol [36] or other polyphenols [37], would accelerate the development of a new treatment.

It is known that the occurrence of NAFLD in humans is strongly associated with obese and diabetic patients [38,39]. A high body mass index (BMI; kg/m^2^), fat mass, and HOMA-IR (insulin resistance index assessed by homeostasis model assessment) were observed in NAFLD patients [23,40]. In addition, plasma parameters such as ALT, AST, glucose, and TG were also high in the NAFLD patients [40,41]. Moreover, the total VLDL-TG secretion rate from the liver is increased in these patients [42]. On the other hand, the plasma T-CHO, LDL, and HDL of these patients are normal or only deviate slightly [40,41]. There are some inconsistencies when integrating these human clinical pathologies with the characteristics of this model. In the 1-week CDAHFD feeding model, body weight did not change the way it did in the ND group, and plasma TG, glucose, T-CHO, HDL, and LDL all decreased, which is consistent with previous findings reported by Tasuda et al. [43]. Additionally, insulin resistance would not occur due to the low blood glucose level in this model. These findings differ from the clinical pathology of NAFLD, which shows the limitations of this model. Mice that are fed a CDAHFD are unable to secrete TG and cholesterol from the liver due to a failure of lipoprotein synthesis by choline deficiency and reduced methionine [44]; therefore, there is decreased T-CHO, HDL, and LDL in the plasma in this model. Future researchers should consider these inconsistencies and mechanisms when using this model. Furthermore, as mentioned earlier, this model showed decreased blood glucose levels. In the qPCR assay, gene expressions of *G6pc* and *Pepck*, which are gluconeogenesis markers, were significantly decreased in the model, which is consistent with the decreased blood glucose level. Inflammation and oxidative stress due to obesity and type 2 diabetes are generally considered to be factors that upregulate gluconeogenesis [45,46]. Therefore, to reveal the mechanisms of a decreased blood glucose level in the model, further detailed analysis focusing on the signaling pathway and metabolite profiling for gluconeogenesis in hepatocytes is required.

In summary, we were able to establish an early-stage NASH model without fibrosis after 1 week of feeding a CDAHFD, which can partially mimic the condition of NASH. Our results can be considered a step forward in revealing the mechanisms and treatments of early-stage irreversible NASH. Moreover, our results involve several important evaluation methods of this model and will thus accelerate research progress.

## 4. Materials and Methods

### 4.1. Animal Experiments

All animal experiments in this study were approved by the Animal Care Committee of the University of Tsukuba (approval number: 20-133). Five-week-old C57/B6J male mice were purchased from the Central Laboratories for Experimental Animals (Tokyo, Japan) and then subjected to a 2-week acclimation period. The mice were bred and maintained in an air-conditioned animal house under specific-pathogen-free (SPF) conditions and subjected to a 12/12 h light and dark cycle. The mice were fed standard mouse pellets and water ad libitum during the acclimation period. At the start of the experiments, the mice were aged 7 weeks. Three types of diets were used in this experiment: ND (Cat#MF; Oriental Yeast, Itabashi, Tokyo, Japan), HFD (Cat#D12492, 60 kcal% fat; Research Diets, New Brunswick, NJ, USA), and CDAHFD (Cat#A06071302, 60 kcal% fat with 0.1% methionine and no added choline; Research Diets). An overview of the animal protocols of these experiments is shown in Figure 1A.

After the acclimation period, the mice were randomly assigned to 3 groups to be fed a ND (*n* = 12), HFD (*n* = 12), or CDAHFD (*n* = 13). There were 4–5 mice/cage, with 3 cages in each group. Food for each diet was provided at 40 g/mouse for 1 week. After 1 week of feeding, the mice were fasted for 5 h. Then, the blood glucose was measured using whole blood from the tail tip. The body weight and food intake were also measured. After that, the mice were euthanized by total blood sampling using an anticoagulant (EDTA-2Na) and cervical dislocation under general anesthesia by inhalation of isoflurane. The whole blood was quickly placed on ice. Dissections were performed to harvest the liver tissues to measure the liver weight, and then the tissues for mitochondrial isolation were placed on ice. The tissues were immersed in 10% Formalin Neutral Buffer Solution to make FFPE tissue blocks, after which they were immersed into liquid nitrogen for the other analyses. The whole blood was centrifuged at 1000× *g* for 10 min at 4 °C, and then aliquots of the plasmas were harvested and stored at −80 °C for further analyses.

### 4.2. Mitochondrial Isolation

Mitochondria in the liver tissues were isolated according to the method reported by Clayton and Shadel [47], with minor modifications as follows: 1× MS homogenization buffer containing 210 mM mannitol, 70 mM sucrose, 5 mM Tris-HCl (pH 7.5), 1 mM EDTA (pH 7.5), 1 mM dithiothreitol, and protease inhibitor cocktail (Cat# 25955-24; Nacalai Tesque, Nakagyo, Kyoto, Japan) were applied to the tissues. Then, the liver tissues were homogenized with a buffer (µL):tissue (mg) ratio of 10:1, using Potter-Elvehjem tissue grinders on ice. The homogenates were centrifuged at 1300× *g* at 4 °C for 10 min. The supernatant of 500 µL was placed in a new micro-tube, and centrifugation was performed once more at 12,000× *g* at 4 °C for 15 min, which produced the mitochondrial pellet. After the centrifugation and removal of the supernatant, the mitochondrial pellet was suspended in 500 µL of the 1× MS homogenization buffer and again centrifuged at 12,000× *g* at 4 °C for 15 min (the wash step). After that, the washed mitochondrial pellets were suspended in 500 µL of PBS and subjected to further analysis. In the ND group, the experimental procedure failed in one sample, which became *n* = 11 in the analysis using isolated mitochondria.

### 4.3. Measurements of General Markers in the Plasma

General plasma biomarkers of ALT (IU/L), AST (IU/L), NEFFA (µEq/L), T-CHO (mg/dL), HDL (mg/dL), and LDL (mg/dL) in the plasma were measured as outsourcing by the Oriental Yeast Co., Ltd. (Itabashi, Tokyo, Japan). Plasma TG (mg/dL) was measured as duplicate measurements using a LabAssay Triglyceride kit (FUJIFILM Wako Pure Chemical Corporation, Osaka, Osaka, Japan), according to manufacturer’s instructions.

### 4.4. Tissue Staining

The FFPE block of the liver was sectioned at a thickness of 4 μm. The sections were placed on glass slides and subjected to HE and MT staining, using Mayer’s Hematoxylin Solution and 1% Eosin Y Solution (Muto Pure Chemicals, Bunkyo, Tokyo, Japan) for HE staining and Aniline Blue Solution, Masson Stain Solution B, 0.75% Orange G Solution, and 2.5% Phosphotungstic Acid Solution (Muto Pure Chemicals, Bunkyo, Tokyo, Japan) for MT staining. The staining protocols followed the manufacturer’s instructions of the Muto Pure Chemicals. After staining, their morphology was examined under a model BZ-X710 microscope (Keyence, Osaka, Osaka, Japan) to assess the lipid droplets, inflammatory findings, and the degree of fibrosis. The lipid droplet areas (%) of the entire image were quantified in 3 different places on the same sections stained by HE as triplicate measurements in ImageJ Fiji (version Java 8). 

The SAF scoring system to elevate the pathology of NAFLD was also applied to the stained tissues, in accordance with previous research [22]. This evaluation was performed by a clinical technologist and medical doctor in duplicate measurements.

IF staining was also conducted to assess the localization signals on the cell accumulation site. The sections on the glass slide were deparaffinized and hydrated. After that, the sections were placed in a 0.01 M citrate buffer (pH 6.0) and subjected to antigen activation at 121 °C for 10 min. Then, the sections were gently washed with phosphate-buffered saline (PBS) for 10 min 3 times and blocked with 5% skim milk/0.3% Triton X-100/PBS (PBS-T) for 1 h at room temperature. After that, they were washed 3 times with PBS for 10 min each, then incubated with the primary antibodies rabbit anti-myeloperoxidase (Cat#EPR20257; Abcam, Cambridge, UK) and mouse anti-α-tubulin (Cat#66031-1-Ig; Proteintech, Rosemont, IL, USA), diluted at 1:100 in 1% BSA/PBS-T in a wet box at 4 °C overnight. After washing with PBS 3 times for 10 min each time, the secondary antibodies goat anti-mouse IgG, FITC conjugate (Cat# SA00003-1; Proteintech), and goat anti-rabbit IgG, TRITC conjugate (Cat# SA00007-2; Proteintech) were diluted at 1:100 in a 1% BSA/PBS-T buffer, applied to the sections, and incubated in a wet box at room temperature for 1 h. After washing three times in PBS for 10 min each, a mounting medium with DAPI (Dapi-Fluoromount-G; Cat#0100-01; SouthernBiotech, Birmingham, AL, USA) was applied to the sections, and they were sealed with a cover glass. The morphology was examined under a model BZ-X710 microscope (Keyence, Osaka, Osaka, Japan) to assess localizations of the nucleus and myeloperoxidase.

### 4.5. Measurements of TG in the Liver

The Folch method [48] was performed on the liver tissues by reference to the textbook [49], with some modifications to extract the lipids. The liver tissues were homogenized in Milli-Q water (Merck Millipore, Burlington, MA, USA) at a ratio of 1:10 (tissue mg:Milli-Q water µL) with zirconia beads, and then 440 µL of the homogenates were transferred to new micro-tubes containing a 1.1 mL chloroform–methanol solution (ratio 2:1). After shaking violently, centrifugation was performed at 15,000× *g* at 4 °C for 5 min. After that, 400 μL of the lower organic layer was transferred to a new micro-tube, and then the solutions were incubated to volatilize on a heat bloc at 80 °C for 10 min. The extracted lipid was dissolved to 100 µL of 5% BSA/0.05% Triton-x/PBS buffer with sonication. TG (mg/dL) was measured by a LabAssay Triglyceride kit (FUJIFILM Wako Pure Chemical Corporation) using 5 µL lipid samples as duplicate measurements. Finally, the quantified values were converted to TG mg/g liver.

### 4.6. TBARS Assay

The TBARS assay was performed according to the method previously reported by Kikugawa [50], with some modifications. This was carried out to measure lipid peroxidation in the liver tissues. First, the TBARS reaction mixture was made by adding the following solutions to one bottle: 2 mL of 5.2% sodium dodecyl sulfate (SDS) in Milli-Q water, 15 mL of a 0.8% solution of thiobarbituric acid (TBA) in Milli-Q Water, 19.125 mL of Milli-Q water alone, 500 µL of a 0.8% solution of butylated hydroxytoluene (BHT) in glacial acetic acid, and 8 mL of a 0.1 M acetate buffer (pH 3.5). The total volume of the mixture was then 42.75 mL. After that, the TBARS mixture and liver tissues were mixed at a ratio of 9:1 (TBARS mixture µL:liver mg) in a micro-tube, and the tissues were homogenized under a bead crusher with zirconia beads. We transferred 500 µL of the homogenate to a new micro-tube, and the samples were incubated at 4 °C for one hour, followed by incubation at 95 °C for 1 h. After cooling the samples to room temperature, 500 μL of 15:1 *v*/*v* 1-butanol and pyridine was added to the chilled tubes, after which they were shaken. The mixtures were centrifuged at 3000 rpm for 10 min at 4 °C. The fluorescence of the supernatant was measured at 540 nm excitation and 590 nm emission. The TBARS concentrations (nmol/mL) were calculated from a regression equation plotted with a 1,1,3,3-tetraethoxypropane standard (200 to 0.4 nmol/mL). Finally, the quantified values were converted to TBARS nmol/g liver. This assay was conducted in duplicate measurements.

### 4.7. DCF Assay

DCF assays were performed to measure the production of free radicals in the liver. This was carried out by referring to the method reported by Ali et al. [51], Puntel et al. [52], and Sugasawa et al. [53], with some modifications, adapted to the liver homogenate. First, the liver tissues (approximately 100 mg) were homogenized with a bead crusher in a protein lysis buffer (1% NP40, 150 mM NaCl, 50 mM Tris-HCl (pH 7.5)) that included a protease inhibitor cocktail (Cat# 25955-24; Nacalai Tesque) in a micro-tube. Then, the homogenates were centrifuged at 12,000× *g* at 4 °C for 15 min. The supernatants of the protein lysate were transferred to new micro-tubes, and the protein concentrations were measured using a BCA assay kit (Cat#RR036A, Takara Bio, Kusatsu, Shiga, Japan) in accordance with the manufacturer’s instructions. After diluting and adjusting the protein lysate to 0.1 mg/mL protein, 10 µL of the protein lysate was mixed with 100 μL of 10 mM Tris-HCl (pH 7.4) containing 5 μM 2′,7′-dichlorodihydrofluorescein diacetate (DCFH-DA) in a black microplate well. After incubating this plate at 37 °C for 60 min, the fluorescence intensity of the solution at 525 nm was measured in duplicate by irradiating it with excitation light at 488 nm. The DCF concentrations were calculated from a regression equation plotted with a DCF standard (200 to 6.25 pmol/mL). Finally, the quantified values were normalized as DCF pmol/mg protein. This assay was conducted using duplicate measurements.

### 4.8. CAT Assay

A CAT assay to measure catalase activity was performed according to the published method by Sigma-Aldrich (St. Louis, MO, USA) [54], with some modifications. First, 10 µL of the adjusted protein lysate (0.1 mg/mL), the same as in the DCF assay (Section 4.7), was mixed with 200 µL of a 50 mM potassium phosphate buffer (pH 7.0) containing 50 mM H_2_O_2_ in a UV microplate, then its absorbance at 240 nm was immediately measured in a microplate reader (0 min). The absorbance at 240 nm was measured again following incubation at room temperature for 10 min. The standard enzyme solution of catalase was also read on the same plate and time. Δ absorbance from 0 min to 10 min was calculated, and then the catalase activity (U/mL) was calculated from a regression equation plotted with the catalase activities standard (10–0.16 U/mL). Finally, the quantified values were normalized as a CAT U/mg protein. This assay was conducted in duplicate measurements.

### 4.9. SOD Assay

A SOD assay to measure the total SOD activity was performed according to the method previously reported by Peskin and Winterbourn [55], with some modifications. First, the following solutions were made: an assay buffer with a 50 mM sodium phosphate buffer (pH 8.0) containing 0.1 mM diethylenetriaminepentaacetic acid (DTPA) and 0.1 mM hypoxanthine, 10 mM WST-1 (DOJINDO, Mashiki, Kumamoto, Japan) in Milli-Q water, catalase (FUJIFILM Wako Pure Chemical Corporation) 2 mg/mL in 50% glycerol, and xanthine oxidase (FUJIFILM Wako Pure Chemical Corporation) 13.8 mg/mL in 50% glycerol. Just before the assay, 20 mL of reaction mixture-1 was made as a 19.88 mL assay buffer/100 µL WST-1 solution/20 µL catalase solution. Additionally, 2 mL of the reaction mixture-2 was made as a 1.98 mL assay buffer/20 µL xanthine oxidase solution. Next, 10 µL of the adjusted protein lysate (0.1 mg/mL), the same as in the DCF assay (Section 4.7), was applied to wells containing 200 µL of the reaction mixture-1 in 96-well clear microplates. After that, reaction mixture-2 was also applied to the wells and mixed, followed by a 20 min incubation at 37 °C. Standard samples to make a standard curve of the SOD activity were also reacted at the same time and way. After the incubation, the absorbance at 450 nm referenced at 600 nm was measured in a microplate reader. Using the standard curve, the SOD activity (U/mL) of each sample was calculated. Finally, the quantified values were normalized as SOD U/mg protein. This assay was conducted in duplicate measurements.

### 4.10. Resazurin Assay for the Mitochondria

The Resazurin assay to measure mitochondrial activity was performed by referring to the method reported by Zhang et al. [56] and James et al. [57], with some modifications to adapt it to the isolated mitochondria from the liver tissues. This assay has been considered able to measure the metabolic activity of mitochondrial respiration and the citric acid cycle [56,57] because Resazurin can react with nicotinamide adenine dinucleotide (NADH) and can be structurally changed as a fluorescence dye to Resorufin. First, Resazurin solution was added to a general cell culture medium (DMEM/high-glucose, continuing antibiotics and 10% FBS) with a concentration of 100 µM to prepare the reaction medium. Then, 20 µL of isolated mitochondrial suspension, intact mitochondria from the liver, in PBS was applied to wells containing 200 µL of the reaction medium in a 96-well clear microplate. After mixing well by pipetting, the plate was incubated in a CO_2_ incubator (5% CO_2_, 37 °C, 100% humidity) for 3 h. After the incubation, 100 µL of the reaction samples were transferred to a 96-well black plate and the fluorescence intensity was measured at 530 nm excitation and 590 nm emission in a microplate reader. Finally, the quantified values were converted to Resorufin fluorescence/mg liver. This assay was conducted as triplicate measurements.

### 4.11. JC-1 Assay for the Mitochondria

The JC-1 assay to measure mitochondrial activity was performed by referring to the method reported by Sugasawa et al. [53], with some modifications, and was adapted to the isolated mitochondria from the liver tissues. First, JC-1 (PromoKine, Sickingenstr, Heidelberg, Germany) dissolved in DMSO was added to a general cell culture medium (DMEM/high-glucose, continuing antibiotics and 10% FBS) with a concentration of 2 µM to prepare the reaction medium. To make a pellet of the mitochondria, 100 µL of the isolated mitochondrial suspension in a micro-tube was centrifuged at 12,000× *g* at 4 °C for 15 min; then, the pellet was resuspended in the reaction medium, followed by incubation at 37 °C for 15 min. After washing with 1 mL of PBS, the stained pellet was suspended in Hank’s Balanced Salt Solution with Mg^2+^, and 100 µL of the suspension was transferred to the 96-well black plates. Subsequently, the fluorescent intensities at an excitation of 535 nm/emission of 595 nm (red fluorescence) were measured in a microplate reader. Finally, the quantified values were converted to JC-1 red fluorescence/mg liver. This assay was conducted in triplicate measurements.

### 4.12. Measurements of mtDNA Copy Numbers in the Isolated Mitochondria

First, 50 µL of isolated mitochondrial suspension was mixed and vortexed with 50 µL of 1% Triton-X/PBS continuing proteinase K (7 U/mL) to make a mitochondrial DNA lysate. The lysate was then incubated at 56 °C for 10 min to degrade the protein, followed by incubation at 95 °C for 10 min to inactivate the proteinase K. After that, the lysate was diluted 10-fold using Milli-Q water. The diluted lysate as a template was subjected to quantitative real-time PCR (qPCR) by SYBR Green dye using a standard curve for absolute quantification of the mitochondrial DNA copy number targeting the *mtCox-1* gene body. The primer sequences are shown in Supplementary Table S1. Detailed conditions of the qPCR are described in Section 4.15. Standard DNA fragments for the standard curve were prepared following gel extraction purification. After the qPCR, it was confirmed that the standard curve was R^2^ = 0.99. Finally, the quantified mitochondrial DNA (mtDNA) copy numbers were converted to mtDNA copy number/mg liver.

### 4.13. Measurements of the mtDNA Copy Numbers in the Liver

To measure the mtDNA copy numbers normalized by genomic DNA (gDNA), the total RNA was extracted from the liver tissues using a phenol/chloroform/isoamyl alcohol solution (Cat#25970-56; Nacalai Tesque). The extraction was performed according to the manufacturer’s instructions. The DNA pellet was dissolved in Milli-Q water, and the DNA concentration was adjusted to 20 ng/µL as the template for the qPCR. The qPCR assay based on SYBR Green dye was performed to target the *mtCox-1* gene body and the gDNA sequence. The primer sequences are shown in Supplementary Table S1. Detailed conditions of the qPCR are described in Section 4.15. After the qPCR assay, mtDNA copy numbers normalized by gDNA (mtDNA/gDNA copy number) were calculated using threshold cycle (CT) values and the 2^ΔΔCT^ method.

### 4.14. Gene Expression Analysis

To quantify gene expression of various markers in the liver and the whole blood, total RNA was extracted from the liver tissues in 50–100 mg and whole blood in 100 µL using Sepasol-RNA I Super G (Cat# 09379-55; Nacalai Tesque). The extraction was performed according to the manufacturer’s instructions. The extracted total RNA solution in Milli-Q water was diluted and adjusted to a concentration of 100 ng/uL. Then, 500 ng of RNA was used to make cDNAs with the PrimeScript RT Master Mix (Cat#RR036A, Takara Bio), according to the manufacturer’s instructions. The cDNAs were diluted at ×10 using Milli-Q water and subjected to qPCR assay based on SYBR Green dye. The target genes were markers for inflammation, cell growth, fibrosis, antioxidative enzymes, NRF2 target genes (reflecting oxidative stress), lipogenesis, and gluconeogenesis. The primer sequences are shown in Supplementary Table S1. Detailed conditions of the qPCR assay are described in Section 4.15. After the qPCR assay, the values of relative gene expression normalized by the *36B4* (*Rplp0*) gene were calculated using threshold cycle (CT) values and the 2^ΔΔCT^ method.

### 4.15. qPCR Assay

First, the reaction plate was prepared as follows: 2 μL of template, 0.1 µL of a 10 µM primer solution (forward and reverse each), 5 µL of master mix (KAPA SYBR FAST qPCR kits, Cat# KK4620; NIPPON Genetics, Bunkyo, Tokyo, Japan), and 2.8 µL of Milli-Q water were included in a total reaction volume of 10 μL per well on a qPCR plate. Negative control wells were also prepared by using Milli-Q water in the assays instead of a template. Then, the qPCR assay was performed on a QuantStudio 5 Real-Time PCR System (Thermo Fisher Scientific, Waltham, MA, USA) in thermal cycle conditions as follows: one cycle of 95 °C for 5 min, followed by 40 cycles of 95 °C for 3 s and 60 °C for 30 s, with a final stage of melting curve analysis. All qPCR assays were conducted as duplicate measurements.

### 4.16. WB Analysis

First, the protein lysate of the whole liver, which is the same sample in the DCF assay (Section 4.7), was adjusted to a protein concentration of 2 mg/mL, and the mitochondrial suspension, which is the same sample in Section 4.2, was prepared. The lysate or suspension was mixed with a 2× loading buffer containing 2-mercaptoethanol and denatured at 95 °C for 5 min. After this procedure, the samples to detect OXPHOS proteins were incubated at room temperature for 20 min instead of at 95 °C for 5 min because the OXPHOS proteins are very sensitive to high temperatures. The 10 µL prepared samples were subjected to sodium dodecyl sulfate polyacrylamide gel electrophoresis (SDS-PAGE) using 10% or 15% gels at 140 V for 70 min, and then the separated proteins in the gel were transferred to the polyvinylidene fluoride (PVDF) membrane using a wet transfer method at 40 V at 4 °C overnight. This membrane was blocked with a TBS-T buffer (50 mM Tris-HCl, 150 mM NaCl, 0.05% Tween 20, pH 7.6) including 5% skim milk for 30 min, and then the membrane was washed with a TBS-T buffer 3 times for a total of 10 min. Subsequently, the membrane was immersed in a properly diluted primary antibody and incubated overnight with gentle shaking at 4 °C. After washing it with a TBS-T buffer 3 times for 10 min, the membrane was immersed in a properly diluted secondary antibody and incubated with gentle shaking for 30 min at room temperature. After washing the membrane, the target protein bands were visualized with a chemiluminescence reagent (Amersham ECL Select, Cat# RPN2235; Cytiva, Shinjuku, Tokyo, Japan) on ImageQuant LAS 4000 (Cytiva), followed by export as 16-bit TIFF images. The luminance of the bands of the TIFF images were quantified using ImageJ Fiji (version Java 8). Information about the antibodies is shown in Supplementary Table S2. Raw data of the WB analysis for all samples are provided in Supplementary Figure S1.

### 4.17. Statistics

All data were statistically analyzed using GraphPad Prism v9.0.2 (GraphPad, San Diego, CA, USA). We conducted the Shapiro–Wilk normality test for all experimental data to check the normality of the distributions. We then decided to use non-parametric tests for all data. Kruskal–Wallis H tests (one-way ANOVA of ranks) were also performed, followed by a two-stage Benjamini, Krieger, and Yekutieli False Discovery Rate (FDR) procedure as a post hoc test. A *p*-value of less than 0.05 was considered statistically significant. In the graphs, individual values were plotted with median and interquartile range. In some graphs, the *y*-axis is displayed logarithmically because there are groups that show drastic changes in the values.

## Data Availability

Not applicable.

## Supplementary Materials

The following are available online at https://www.mdpi.com/article/10.3390/ijms22115851/s1: Figure S1: raw data of WB analysis for all samples, Table S1: primer sequences used in this study, Table S2: information of the antibodies in the WB analysis.
