# Supplementary material for: One Week of CDAHFD Induces Steatohepatitis and Mitochondrial Dysfunction with Oxidative Stress in Liver
Source: Int J Mol Sci. 2021 May 29;22(11):5851. doi: 10.3390/ijms22115851 (PMC8198552; doi:10.3390/ijms22115851)
Supplement: Supplementary file 1 [file ijms-22-05851-s001.zip › Supplementary Figure S1 gene expressions of SOD1 and 2.pdf]

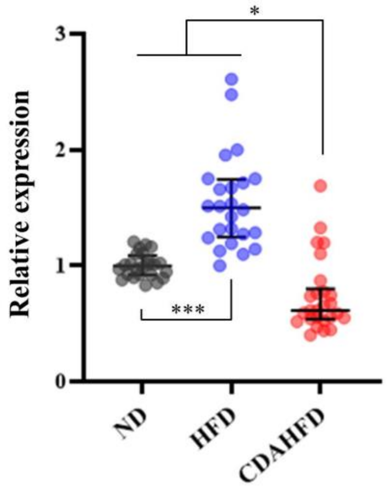

Figure S1. Gene expression analysis for integrated *Sod1* and *2*

The integrated analysis of *Sod1* and *2* in each group showed significant decrease the relative expression values in CDAHFD group compared to other 2 group. On the other hand in the HFD group, the values were increased compared to other 2 group. *$p<0.05$ and ***$p<0.001$, respectively.
